# Supplementary material for: Socio-Demographic Factors and Public Knowledge of Antibiotic Resistance
Source: Healthcare (Basel). 2023 Aug 14;11(16):2284. doi: 10.3390/healthcare11162284 (PMC10454014; doi:10.3390/healthcare11162284)
Supplement: Supplementary file 1 [file healthcare-11-02284-s001.zip › healthcare-2508500-supplementary.pdf]

<u>English Questionnaire</u>

1. Survey Participation Consent
    a. I consent
    b. I do not consent

Section A: Participant Demographics

2. Gender
    a. Male
    b. Female
3. Age (years)
    a. 18-23
    b. 24-29
    c. 30-39
    d. 40-49
    e. 5o-59
    f. 60+
4. Where do you currently reside in Malaysia?
    a. Federal Territory of Kuala Lumpur
    b. Federal Territory of Labuan
    c. Federal Territory of Putrajaya
    d. Johor
    e. Kedah
    f. Kelantan
    g. Malacca
    h. Negeri Sembilan
    i. Pahang
    j. Penang
    k. Perak
    l. Perlis
    m. Sabah
    n. Sarawak
    o. Selangor
    p. Terengganu
5. Which of these best describes where you live?
    a. Urban – within a densely populated city/town
    b. Suburban – in a suburb of a city/town
    c. Rural – outside of a city/town, e.g. village/countryside/farming area
6. What is the highest degree or level of school you have completed? If currently enrolled in a program, please state your previous completed qualification.
    a. No schooling completed
    b. High school or less, no diploma/ qualifications
    c. High school graduate with diploma/ qualifications
    d. Pre-university, no degree (STPM, A-Levels, Matriculation etc.)
    e. Technical/Vocational training or Associate degree
    f. Bachelor's degree
    g. Master's/ Professional degree
    h. Doctorate degree

7. Which of following best describes your monthly household income (RM), before tax?
    a. No income
    b. Less than 2,500
    c. 2,500-4,849
    d. 4,850-7,099
    e. 7,110-10,959
    f. 10,960-15,039
    g. More than 15,040
8. Please select your racial group
    a. Malay
    b. Chinese
    c. Indian
    d. Other:_____
9. Which of these best describes the members in your house including yourself? Adult age is 18 years and above.
    a. 1 Single adult only
    b. 1 Single adult and at least 1 child under 18
    c. Married / live-in partnership - adults only
    d. Married / live-in partnership and at least 1 child under 18
    e. Multiple adults
    f. Multiple adults and at least 1 child under 18

Section B: Use of Antibiotics

10. When did you last take antibiotics?
    a. In the last month
    b. In the last 6 months
    c. In the last year
    d. More than a year ago
    e. Never
    f. Can't remember

    If 'Never', go straight to Question 14.

11. On that occasion, did you get the antibiotics (or a prescription for them) from a doctor or nurse?
    a. Yes
    b. No
    c. Can't remember
12. On that occasion, did you get advice from a doctor, nurse or pharmacist on how to take them?
    a. Yes, I received advice on how to take them (e.g. with food, for 7 days)
    b. No
    c. Can't remember
13. On that occasion, where did you get the antibiotics?
    a. Medical store or pharmacy
    b. Stall or hawker
    c. The internet
    d. Friend or family member
    e. I had them saved up from a previous time

f. Somewhere/someone else
g. Can't remember

Section C: Knowledge about Antibiotics

14. When do you think you should you stop taking antibiotics once you've begun treatment?
    a. When you feel better
    b. When you've taken all of the antibiotics as directed
    c. Don't know
15. "It's okay to use antibiotics that were given to a friend or family member, as long as they were used to treat the same illness"
    a. True
    b. False
    c. Don't know
16. "It's okay to buy the same antibiotics, or request these from a doctor, if you're sick and they helped you get better when you had the same symptoms before."
    a. True
    b. False
    c. Don't know
17. Which of the following conditions can be treated with antibiotics? You may choose more than one answer.
    a. HIV/AIDS
    b. Gonorrhoea
    c. Bladder infection or urinary tract infection (UTI)
    d. Diarrhoea
    e. Cold and flu
    f. Fever
    g. Malaria
    h. Measles
    i. Skin or wound infection
    j. Sore throat
    k. Body aches
    l. Headaches

Section D: Knowledge about antibiotic resistance

18. Have you heard of any of the following terms?
    a. Antibiotic resistance
        i. Yes
        ii. No
    b. Superbugs
        i. Yes
        ii. No
    c. Antimicrobial resistance
        i. Yes
        ii. No
    d. AMR
        i. Yes
        ii. No
    e. Drug resistance

        i. Yes
        ii. No
    f. Antibiotic-resistant bacteria
        i. Yes
        ii. No

19. If you answered YES for question 18) to 'Antibiotic Resistance.' Where did you hear about the term: 'Antibiotic Resistance'? You may skip this question if you answered NO for question 18.
    a. Doctor or nurse
    b. Pharmacist
    c. Family member or friend (including on social media)
    d. Media (newspaper, TV, radio)
    e. Specific campaign
    f. Other
    g. Can't remember
20. If you answered YES for question 18) to ‘Superbugs.' Where did you hear about the term: 'Antibiotic Resistance'? You may skip this question if you answered NO for question 18.
    a. Doctor or nurse
    b. Pharmacist
    c. Family member or friend (including on social media)
    d. Media (newspaper, TV, radio)
    e. Specific campaign
    f. Other
    g. Can't remember
21. If you answered YES for question 18) to ‘Antimicrobial Resistance.’ Where did you hear about the term: 'Antibiotic Resistance'? You may skip this question if you answered NO for question 18.
    a. Doctor or nurse
    b. Pharmacist
    c. Family member or friend (including on social media)
    d. Media (newspaper, TV, radio)
    e. Specific campaign
    f. Other
    g. Can't remember
22. If you answered YES for question 18) to ‘AMR.' Where did you hear about the term: 'Antibiotic Resistance'? You may skip this question if you answered NO for question 18.
    a. Doctor or nurse
    b. Pharmacist
    c. Family member or friend (including on social media)
    d. Media (newspaper, TV, radio)
    e. Specific campaign
    f. Other
    g. Can't remember
23. If you answered YES for question 18) to ‘Drug Resistance.' Where did you hear about the term: 'Antibiotic Resistance'? You may skip this question if you answered NO for question 18.
    a. Doctor or nurse
    b. Pharmacist
    c. Family member or friend (including on social media)

d. Media (newspaper, TV, radio)
e. Specific campaign
f. Other
g. Can't remember

24. If you answered YES for question 18) to ‘Antibiotic-resistant bacteria.' Where did you hear about the term: 'Antibiotic Resistance'? You may skip this question if you answered NO for question 18.
    a. Doctor or nurse
    b. Pharmacist
    c. Family member or friend (including on social media)
    d. Media (newspaper, TV, radio)
    e. Specific campaign
    f. Other
    g. Can't remember

Section E: Participant opinions on antibiotic resistance

25. Please indicate whether you think the following statements are 'true' or 'false'

	True	False
Antibiotic resistance occurs when your body becomes resistant to antibiotics and they no longer work as well		
Many infections are becoming increasingly resistant to treatment by antibiotics		
If bacteria are resistant to antibiotics, it can be very difficult or impossible to treat the infections they cause		
Antibiotic resistance is an issue that could affect me or my family		
Antibiotic resistance is an issue in other countries but not here		
Antibiotic resistance is only a problem for people who take antibiotics regularly		
Bacteria which are resistant to antibiotics can be spread from person to person		
Antibiotic-resistant infections could make medical procedures like surgery, organ transplants and cancer treatment much more dangerous		

26. On the scale shown, how much do you agree the following actions would help address the problem of antibiotic resistance?

	5- Agree Strongly	4- Agree Slightly	3- Neither agree nor disagree	2- Disagree Slightly	1-Disagree Strongly
People should use antibiotics only when they are prescribed by a doctor or nurse					

Farmers should give fewer antibiotics to food-producing animals					
People should not keep antibiotics and use them later for other illnesses					
Parents should make sure all of their children's vaccinations are up-to-date					
People should wash their hands regularly					
Doctors should only prescribe antibiotics when they are needed					
Governments should reward the development of new antibiotics					
Pharmaceutical companies should develop new antibiotics					

27. On the scale shown, how much do you agree with following statements?

	5- Agree Strongly	4- Agree Slightly	3- Neither agree nor disagree	2- Disagree Slightly	1-Disagree Strongly
Antibiotic resistance is one of the biggest problems the world faces					
Medical experts will solve the problem of antibiotic					

resistance before it becomes too serious					
Everyone needs to take responsibility for using antibiotics responsibly					
There is not much people like me can do to stop antibiotic resistance					
I am worried about the impact that antibiotic resistance will have on my health, and that of my family					
I am not at risk of getting an antibiotic-resistant infection, as long as I take my antibiotics correctly.					

Section F: Use of Antibiotics in Agriculture

28. Do you think antibiotics are widely used in agriculture (including in food-producing animals) in your country?
    a. Yes
    b. No
    c. Don't know

Bahasa Melayu Questionnaire

1. Persetujuan Penyertaan Kajian
    c. Saya setuju
    d. Saya tidak bersetuju

Bahagian A: Demografik Responden

2. Jantina
    a. Lelaki
    b. Perempuan
3. Umur (tahun)
    a. 18-23
    b. 24-29
    c. 30-39
    d. 40-49
    e. 50-59
    f. 60+
4. Di manakah anda tinggal di Malaysia sekarang?
    a. Johor
    b. Kedah
    c. Kelantan
    d. Melaka
    e. Negeri Sembilan
    f. Pahang
    g. Perak
    h. Perlis
    i. Pulau Pinang
    j. Sabah
    k. Sarawak
    l. Selangor
    m. Terengganu
    n. Wilayah Persekutuan Kuala Lumpur
    o. Wilayah Persekutuan Labuan
    p. Wilayah Persekutuan Putrajaya
5. Manakah antara berikut yang paling sesuai menerangkan tempat tinggal anda?
    a. Bandar - dalam tempat yang padat dengan penduduk
    b. Pinggir bandar - daerah terpencil bandar, terutamanya kawasan kediaman
    c. Luar bandar - di luar bandar, contoh: kawasan kampung/ ladangUrban – within a densely populated city/town
6. Apakah ijazah atau tahap tertinggi sekolah yang telah anda selesaikan? Jika anda sedang melaksanakan program pada masa ini, sila nyatakan kelayakan anda sebelum ini.
    a. Tiada persekolahan selesai
    b. Sekolah menengah atau kurang, tiada diploma/kelayakan
    c. Lulusan sekolah menengah dengan diploma/kelayakan
    d. Pra-universiti, tiada ijazah (STPM, A-Levels, Matrikulasi dll.)
    e. Latihan Teknikal/Vokasional atau ijazah Bersekutu
    f. Ijazah Sarjana Muda
    g. Ijazah Sarjana/ Profesional

    h. Ijazah kedoktoran
7. Pendapatan isi rumah bulanan (RM)* Tanpa mengira cukai
    a. Tiada pendapatan
    b. Kurang dari 2,500
    c. 2,500-4,849
    d. 4,850-7,099
    e. 7,110-10,959
    f. 10,960-15,039
    g. Lebih dari 15,040
8. Sila pilih kaum anda
    a. Melayu
    b. Cina
    c. India
    d. Other:_____
9. Manakah antara ini menggambarkan ahli di rumah anda termasuk diri anda sendiri? Umur dewasa ialah 18 tahun ke atas.
    a. 1 dewasa bujang sahaja
    b. 1 dewasa bujang dan sekurang-kurangnya 1 kanak-kanak di bawah umur 18 tahun
    c. Pasangan berkahwin/ tinggal bersama- dewasa sahaja
    d. Pasangan berkahwin/ tinggal bersama dengan sekurang-kurangnya 1 kanak-kanak di bawah umur 18 tahun
    e. Beberapa orang dewasa
    f. Beberapa orang dewasa dan sekurang-kurangnya 1 kanak-kanak di bawah umur 18 tahun

Bahagian B: Kegunaan antibiotik

10. Bilakah kali terakhir anda mengambil antibiotik?
    a. Pada bulan lepas
    b. Dalam 6 bulan lepas
    c. Pada tahun lepas
    d. Lebih setahun yang lalu
    e. Tidak pernah
    f. Saya tidak ingat

    Kalau ‘Tidak pernah’, langkau ke soalan 14.

11. Pada masa itu, adakah anda mendapat antibiotik (atau preskripsi untuk mereka) daripada doktor atau jururawat?
    a. Ya
    b. Tidak
    c. Saya tidak ingat
12. Pada masa itu, adakah anda mendapat nasihat daripada doktor, jururawat atau ahli farmasi tentang cara mengambilnya?
    a. Ya, saya menerima nasihat tentang cara mengambil antibiotik (cth. dengan makanan, selama 7 hari)
    b. Tidak
    c. Saya tidak ingat
13. Pada masa itu, di manakah anda mendapat antibiotik?
    a. Kedai perubatan atau farmasi

b. Gerai atau penjaja
c. Laman web
d. Kawan atau ahli keluarga
e. Saya telah simpan dari masa lepas
f. Dari tempat lain/ orang lain
g. Saya tidak ingat

Bahagian C: Pengetahuan tentang antibiotik

14. Pada pendapat anda, bilakah anda perlu berhenti mengambil antibiotik sebaik sahaja anda memulakannya?
    a. Apabila anda berasa sihat
    b. Apabila anda telah mengambil semua antibiotik yang diberikan
    c. Saya tidak tahu
15. "Tidak mengapa untuk mengambil antibiotik yang diberikan kepada rakan atau ahli keluarga, asalkan ia digunakan untuk merawat penyakit yang sama"
    a. Benar
    b. Tidak benar
    c. Saya tidak tahu
16. "Tidak mengapa untuk membeli/meminta antibiotik yang sama, atau meminta ini dari doktor, jika anda sakit dan mereka membantu anda sembuh apabila anda mengalami simptom yang sama sebelum ini"
    a. Benar
    b. Tidak benar
    c. Saya tidak tahu
17. Manakah antara keadaan berikut boleh dirawat dengan antibiotik? * Anda boleh memilih lebih daripada satu jawapan.
    a. HIV/AIDS
    b. Gonorea
    c. Jangkitan pundi kencing atau jangkitan saluran kencing (UTI)
    d. Cirit-birit
    e. Selesema
    f. Demam
    g. Malaria
    h. Campak
    i. Jangkitan kulit atau luka
    j. Sakit tekak
    k. Sakit badan
    l. Sakit kepala

Bahagian D: Pengetahuan tentang rintangan antibiotik

18. Pernahkah anda mendengar mana-mana istilah berikut?
    a. Rintangan antibiotik (Antibiotic resistance)
        i. Ya
        ii. Tidak
    b. “Superbugs”
        i. Ya
        ii. Tidak

c. Rintangan antimicrobial (Antimicrobial resistance)
    i. Ya
    ii. Tidak
d. AMR
    i. Ya
    ii. Tidak
e. Rintangan ubat (Drug resistance)
    i. Ya
    ii. Tidak
f. Bakteria rintangan antibiotik (Antibiotic-resistant bacteria)
    i. Ya
    ii. Tidak

19. Jika anda menjawab YA untuk soalan 18) 'Rintangan Antibiotik' atau "Antibiotic resistance." Di manakah anda mendengar tentang istilah: 'Rintangan Antibiotik' atau "Antibiotic Resistance?" Anda boleh melangkau soalan ini jika anda menjawab TIDAK untuk soalan 18
    a. Doktor atau jururawat
    b. Ahli farmasi
    c. Ahli keluarga atau rakan (termasuk di media sosial)
    d. Media (akhbar, TV, radio)
    e. Kempen khusus
    f. Lain-lain
    g. Saya tidak ingat
20. Jika anda menjawab YA untuk soalan 18) “Superbugs" Di manakah anda mendengar tentang istilah: “Superbugs”? Anda boleh melangkau soalan ini jika anda menjawab TIDAK untuk soalan 18
    a. Doktor atau jururawat
    b. Ahli farmasi
    c. Ahli keluarga atau rakan (termasuk di media sosial)
    d. Media (akhbar, TV, radio)
    e. Kempen khusus
    f. Lain-lain
    g. Saya tidak ingat
21. Jika anda menjawab YA untuk soalan 18) Rintangan antimikrobial atau "Antimicrobial Resistance." Di manakah anda mendengar tentang istilah: Rintangan antimikrobial atau "Antimicrobial Resistance?" Anda boleh melangkau soalan ini jika anda menjawab TIDAK untuk soalan 18
    a. Doktor atau jururawat
    b. Ahli farmasi
    c. Ahli keluarga atau rakan (termasuk di media sosial)
    d. Media (akhbar, TV, radio)
    e. Kempen khusus
    f. Lain-lain
    g. Saya tidak ingat
22. Jika anda menjawab YA untuk soalan 18) ‘AMR.’ Di manakah anda mendengar tentang istilah: AMR? Anda boleh melangkau soalan ini jika anda menjawab TIDAK untuk soalan 18
    a. Doktor atau jururawat
    b. Ahli farmasi
    c. Ahli keluarga atau rakan (termasuk di media sosial)

    d. Media (akhbar, TV, radio)
    e. Kempen khusus
    f. Lain-lain
    g. Saya tidak ingat

23. Jika anda menjawab YA untuk soalan 18) Rintangan ubat atau "Drug Resistance." Di manakah anda mendengar tentang istilah: Rintangan ubat atau "Drug resistance?" Anda boleh melangkau soalan ini jika anda menjawab TIDAK untuk soalan 18
    a. Doktor atau jururawat
    b. Ahli farmasi
    c. Ahli keluarga atau rakan (termasuk di media sosial)
    d. Media (akhbar, TV, radio)
    e. Kempen khusus
    f. Lain-lain
    g. Saya tidak ingat
24. Jika anda menjawab YA untuk soalan 18) Bakteria rintangan antibiotik atau "Antibiotic-resistant bacteria." Di manakah anda mendengar tentang istilah: Bakteria rintangan antibiotik atau "Antibiotic-resistant bacteria?" Anda boleh melangkau soalan ini jika anda menjawab TIDAK untuk soalan 18
    a. Doktor atau jururawat
    b. Ahli farmasi
    c. Ahli keluarga atau rakan (termasuk di media sosial)
    d. Media (akhbar, TV, radio)
    e. Kempen khusus
    f. Lain-lain
    g. Saya tidak ingat
25. Sila nyatakan sama ada anda fikir pernyataan berikut adalah 'benar' atau 'tidak benar'

	Benar	Tidak Benar
Rintangan antibiotik berlaku apabila badan telah mula mengalami rintangan terhadap antibiotik dan tidak lagi berfungsi dengan baik		
Banyak jangkitan telah memberi rintangan terhadap rawatan antibiotik.		
Jika berlaku rintangan bakteria. ia boleh menjadi sukar untuk merawat jangkitan kuman.		
Rintangan antibiotik ialah isu yang boleh menjejaskan saya atau keluarga saya		
Rintangan antibiotik adalah isu di negara lain dan bukan di negara Malaysia.		
Rintangan antibiotik hanya menjadi masalah bagi orang yang mengambil antibiotik dengan kerap		
Bakteria yang mempunyai rintangan terhadap antibiotik boleh merebak dari seorang ke seorang		
Jangkitan yang mempunyai rintangan antibiotik boleh merumitkan dan membahayakan prosedur perubatan seperti pembedahan. pemindahan organ dan rawatan kanser		

Bahagian E: Pendapat responden tentang rintangan antibiotik

26. Pada skala yang ditunjukkan, sejauh manakah anda bersetuju tindakan berikut akan membantu menangani masalah rintangan antibiotik?

	5- Sangat Setuju	4- Setuju	3- Neutral	2- Tidak bersetuju sedikit	1-Sangat tidak bersetuju
Kita harus menggunakan antibiotik hanya apabila ia dinasihati oleh doktor atau jururawat					
Petani harus memberi sedikit antibiotik kepada haiwan yang menghasilkan makanan					
Kita tidak boleh menyimpan antibiotik menggunakannya untuk penyakit lain					
Ibu bapa harus memastikan vaksinasi anak-anak telah lengkap mengikut jadual imunisasi					
Kita hendaklah kerap mencuci tangan					
Doktor hanya perlu memberikan antibiotik apabila ia diperlukan					
Kerajaan harus memberi ganjaran kepada penghasilan antibiotik baharu					
Syarikat farmaseutikal harus menghasilkan antibiotik baharu					

27. Pada skala yang ditunjukkan, sejauh manakah anda bersetuju dengan pernyataan berikut?

	5- Sangat Setuju	4- Setuju	3- Neutral	2- Tidak bersetuju sedikit	1-Sangat tidak bersetuju
Rintangan antibiotik adalah salah masalah satu terbesar yang dihadapi dunia					
Pakar perubatan akan menyelesaikan masalah rintangan antibiotik sebelum ia menjadi terlalu serius					
Setiap orang perlu bertanggungjawab menggunakan antibiotik secara betul					
Orang seperti saya tidak dapat membuat apa-apa untuk menghentikan rintangan antibiotik					
Saya bimbang tentang kesan rintangan antibiotic terhadap kesihatan saya dan keluarga saya					
Saya tidak berisiko mendapat jangkitan rintangan antibiotik. selagi saya mengambil antibiotik dengan betul.					

Bahagian F: Pengunaan Antibiotik dalam Pertanian

28. Pada pendapat anda, adakah antibiotik digunakan secara meluas dalam sektor pertanian (termasuk penternakan haiwan) di negara anda?
    a. Ya
    b. Tidak

c. Saya tidak tahu
